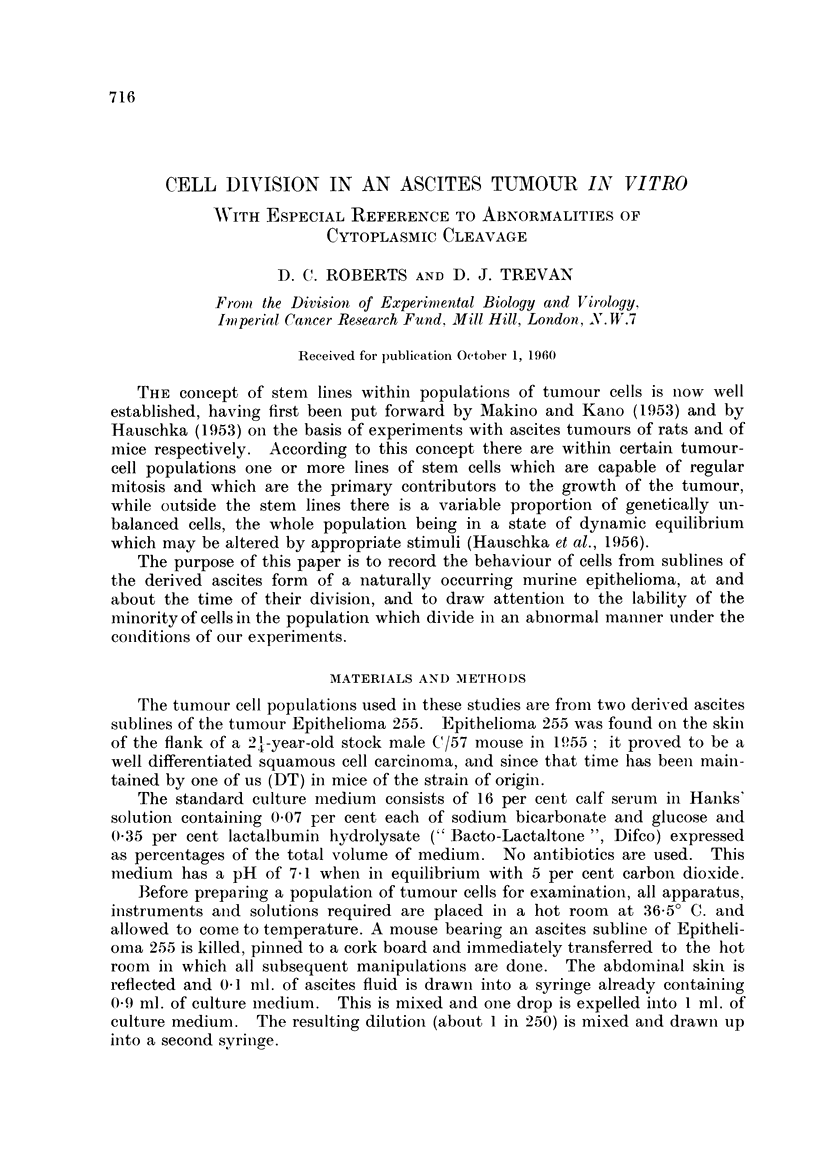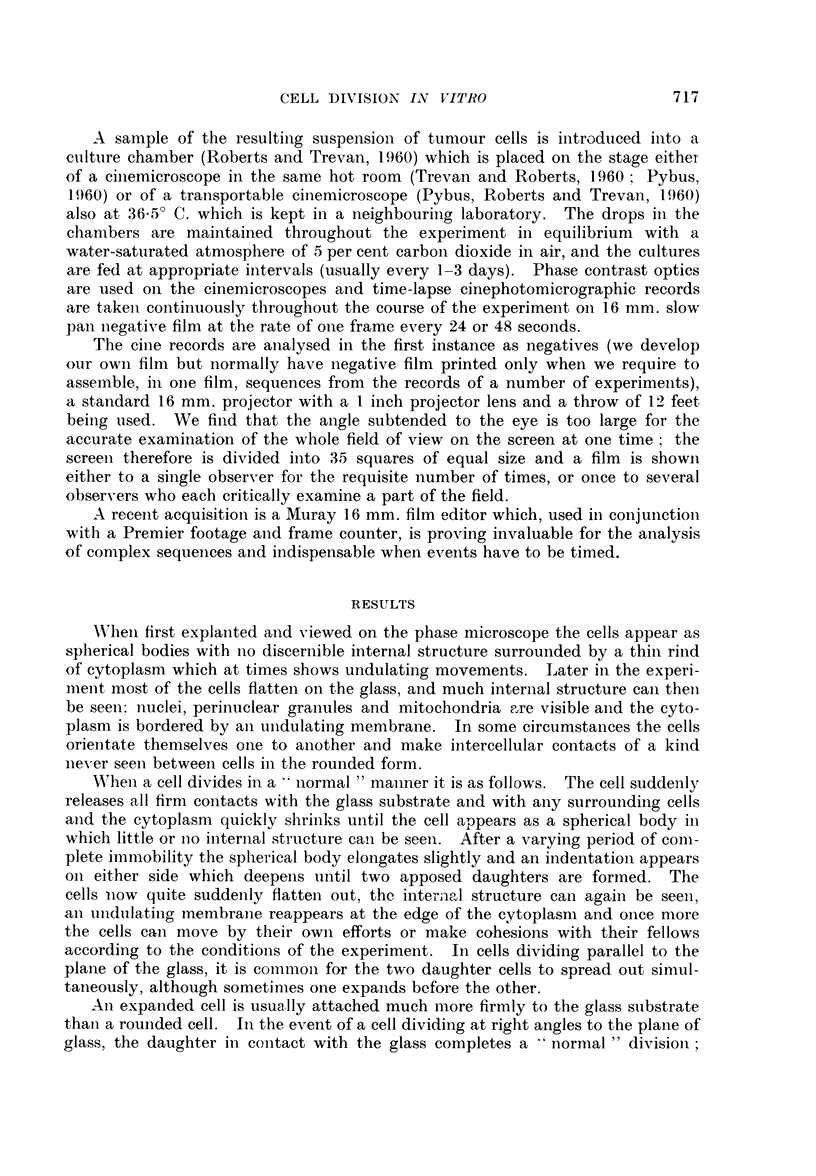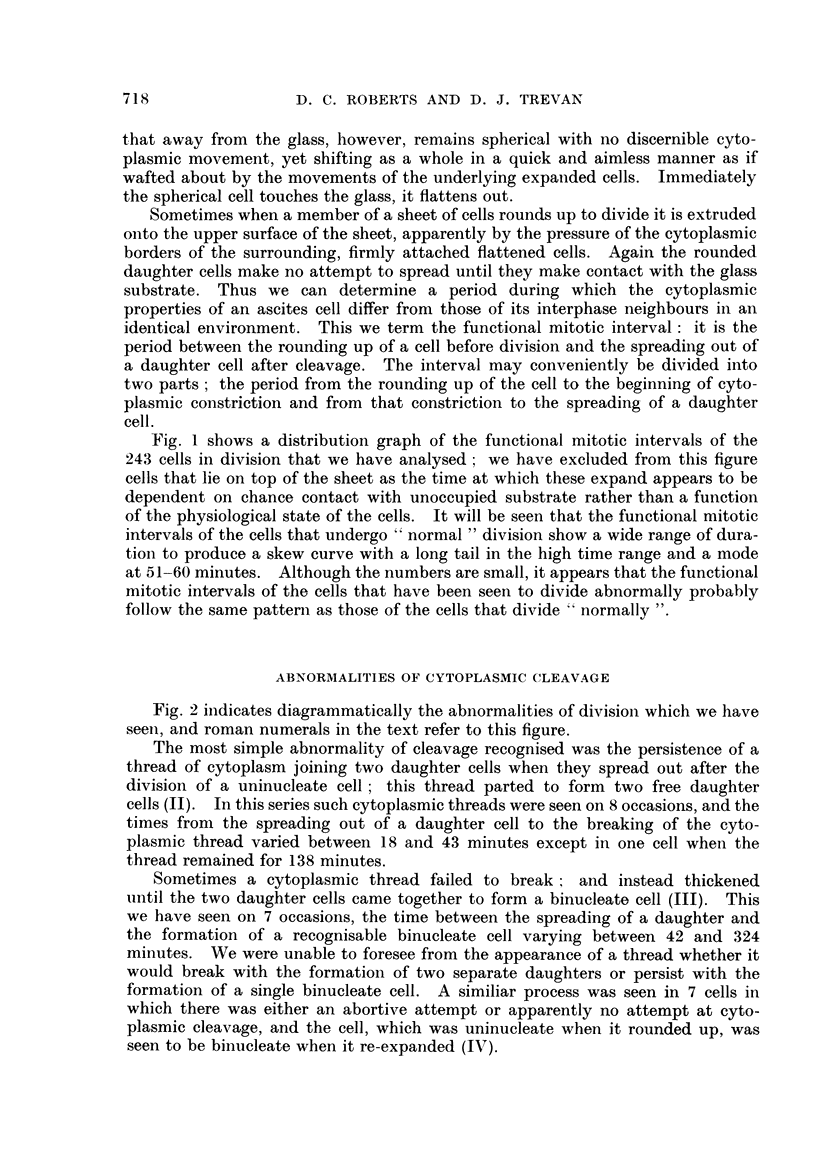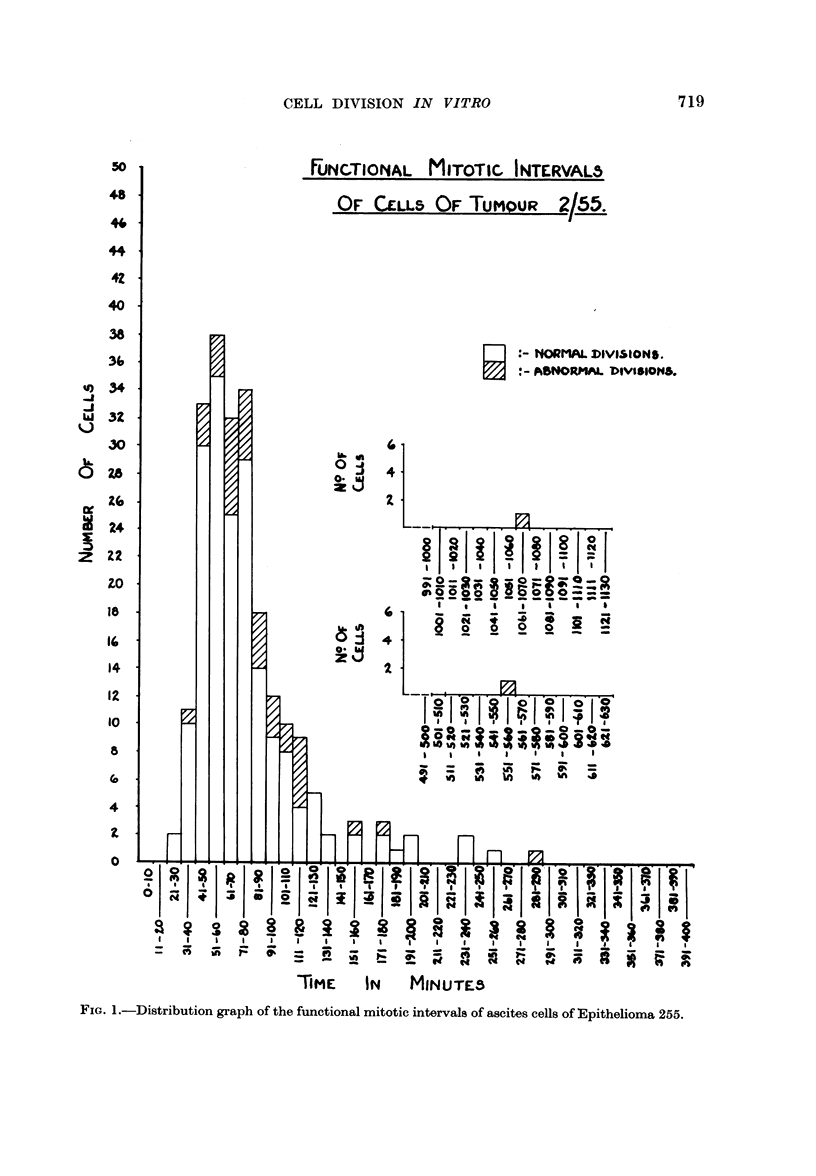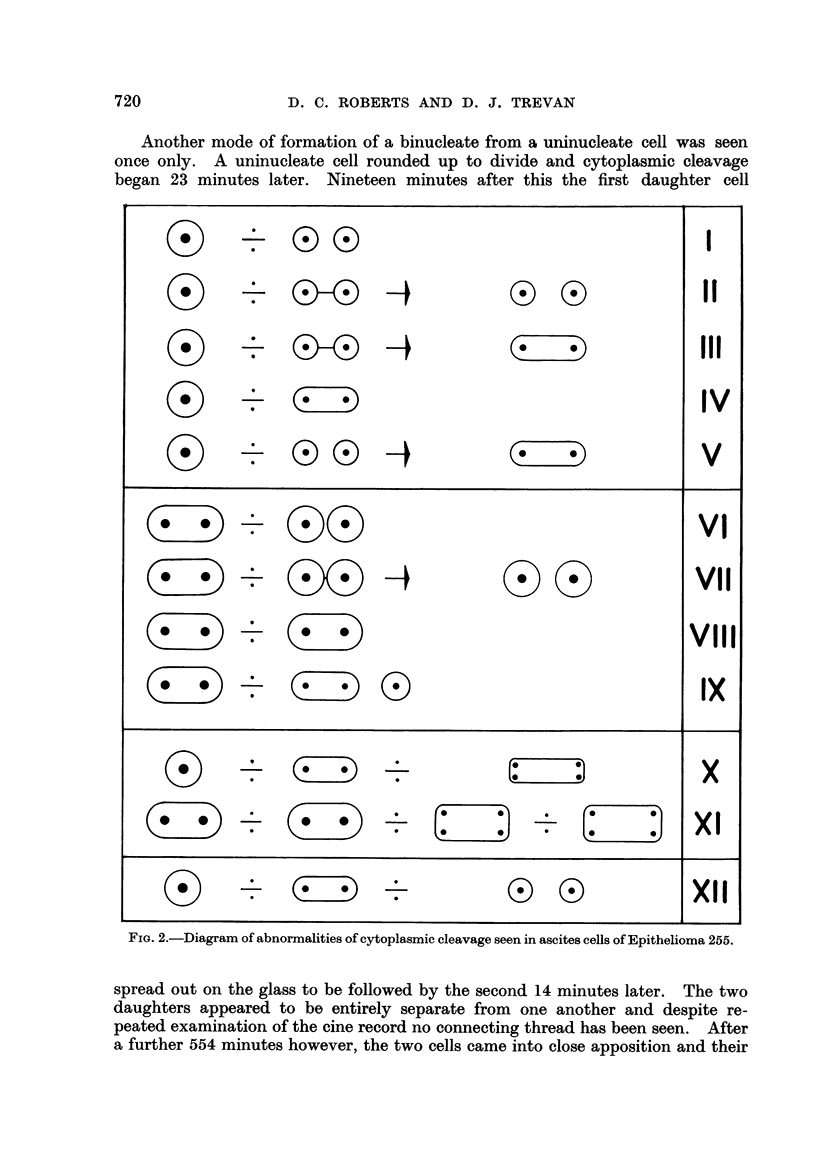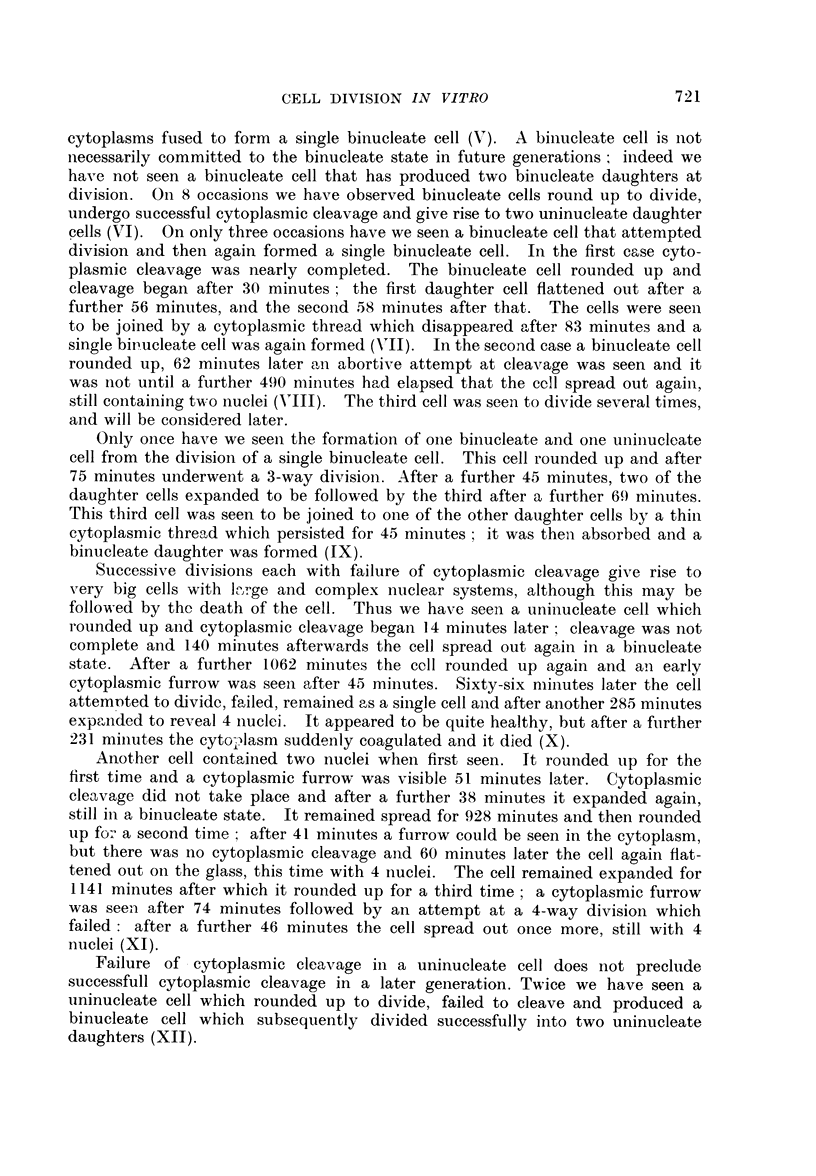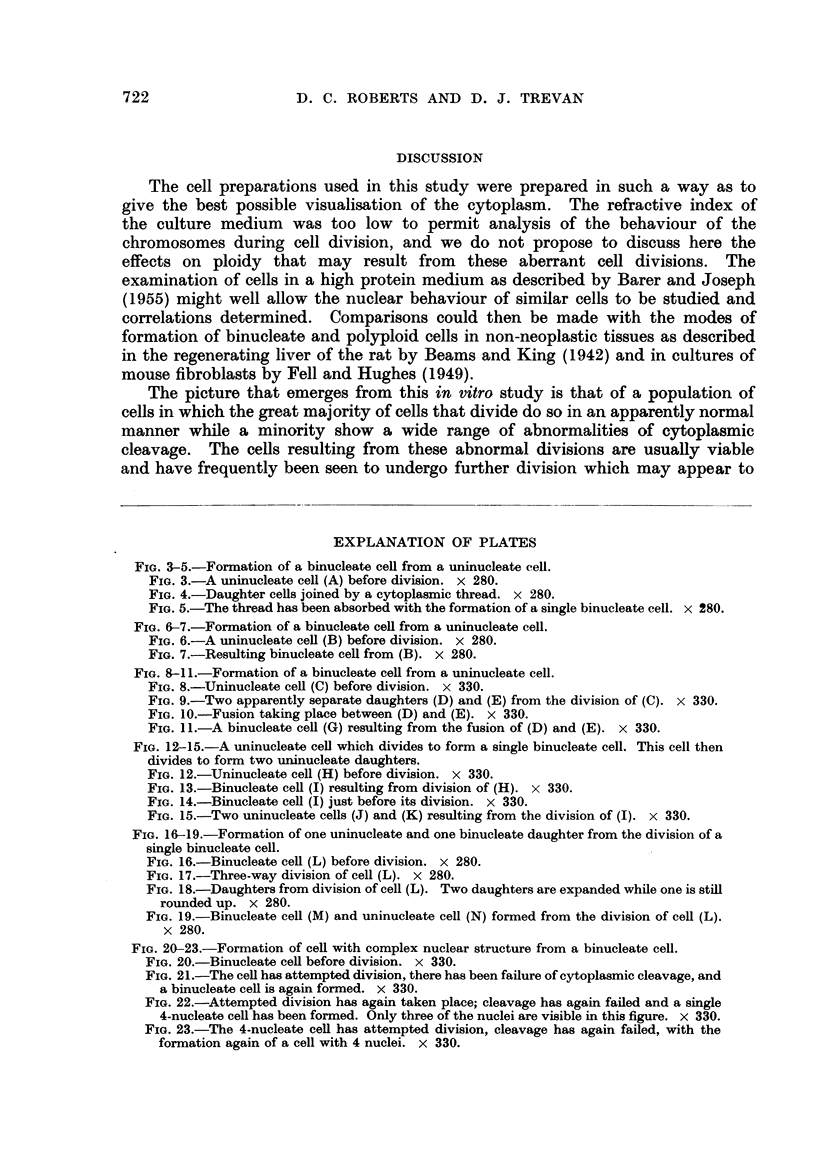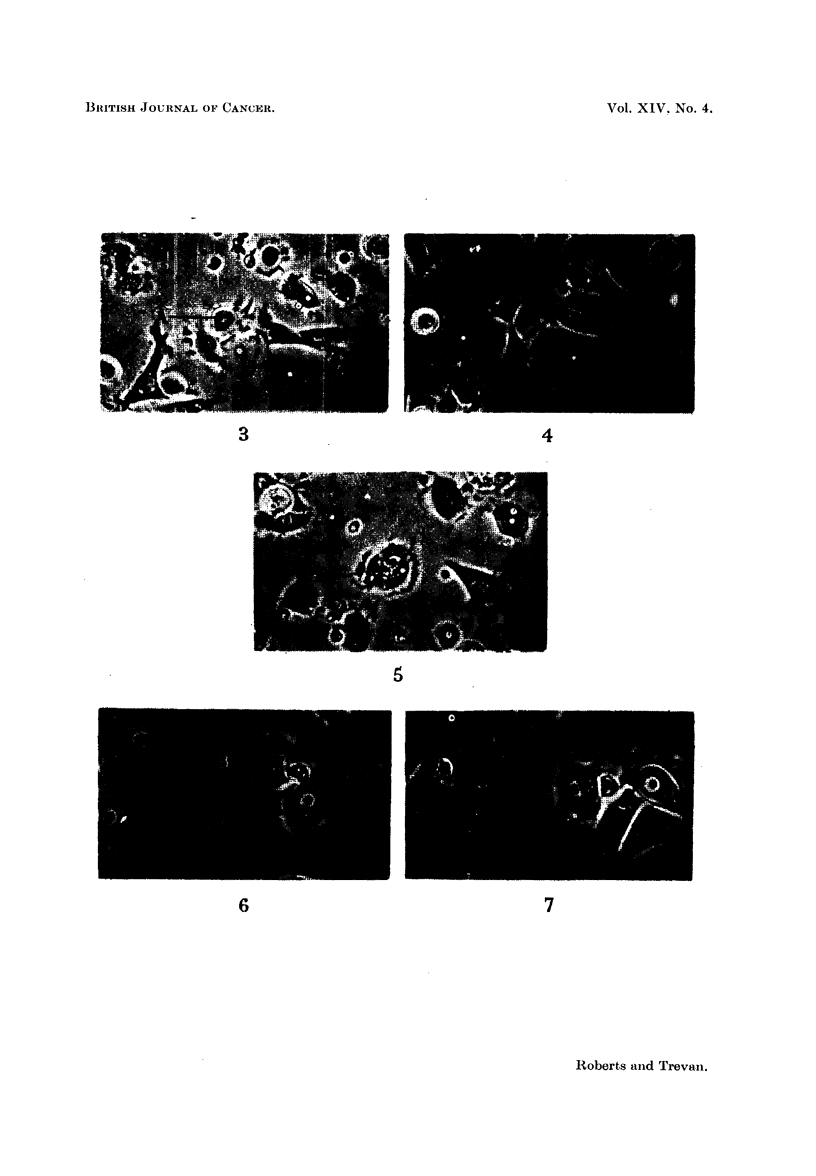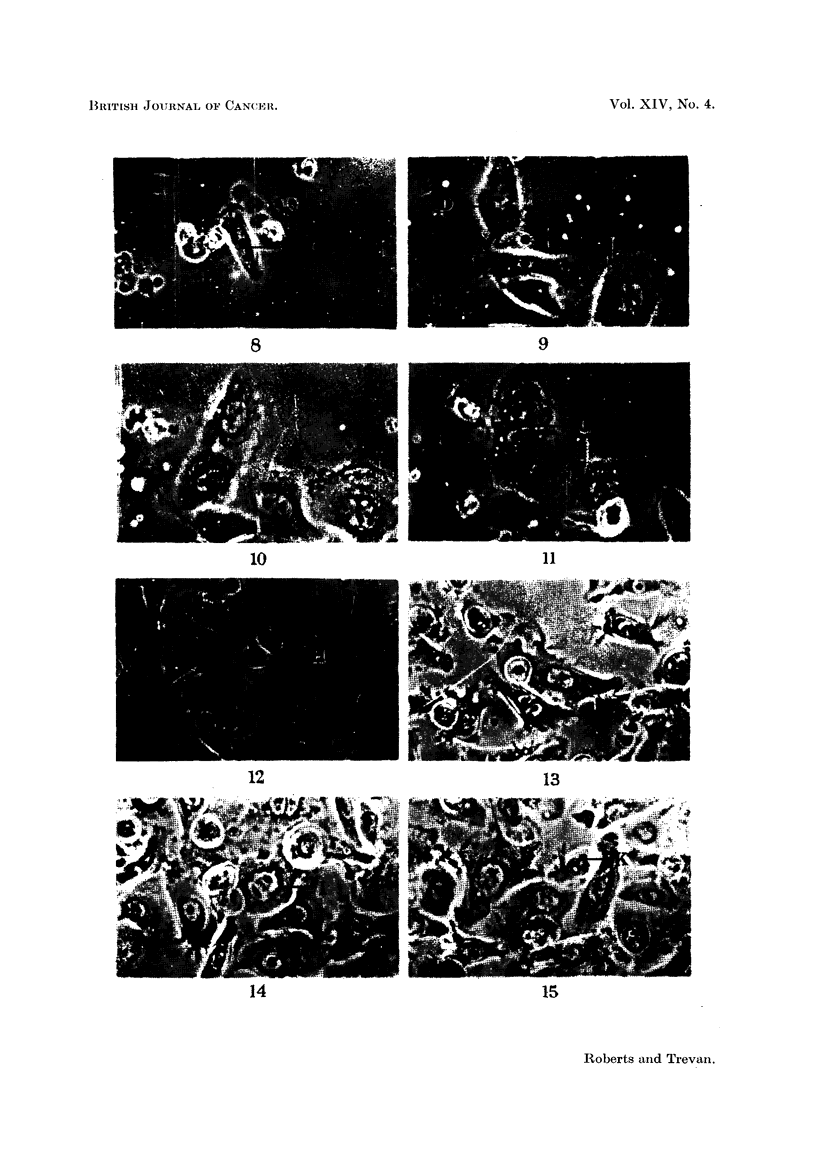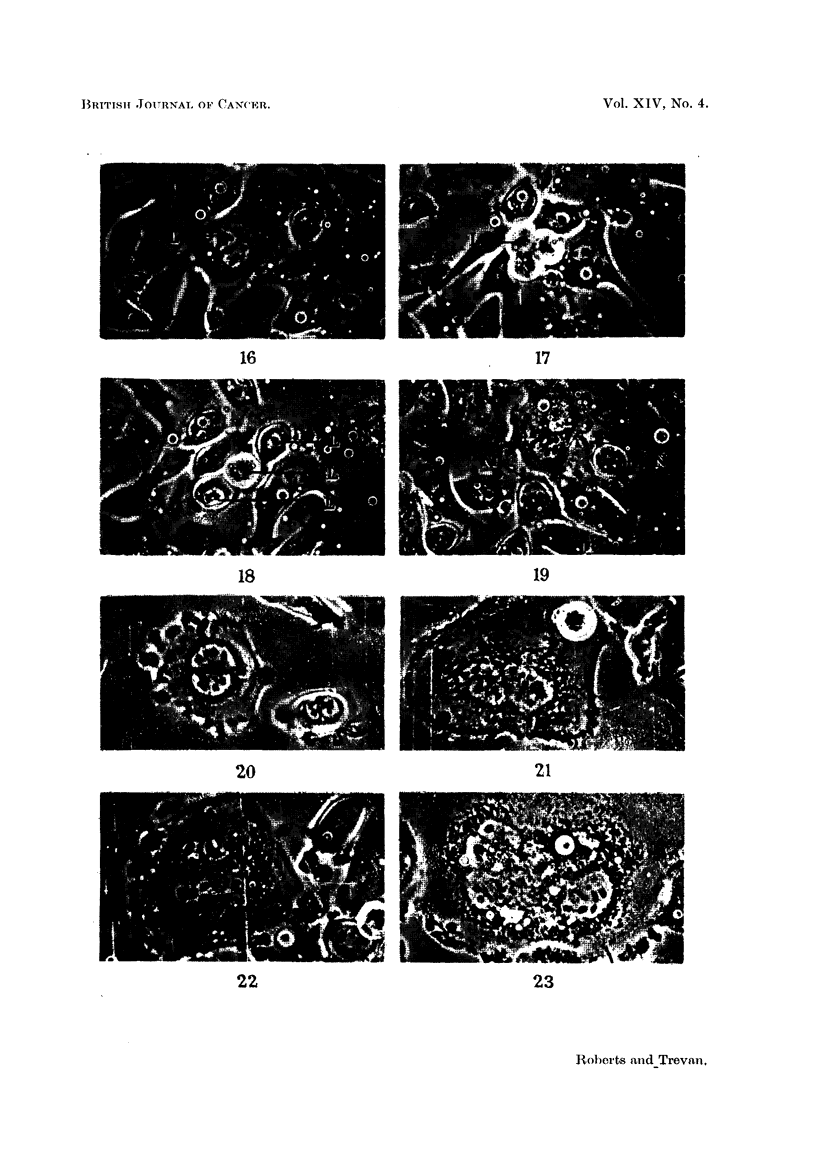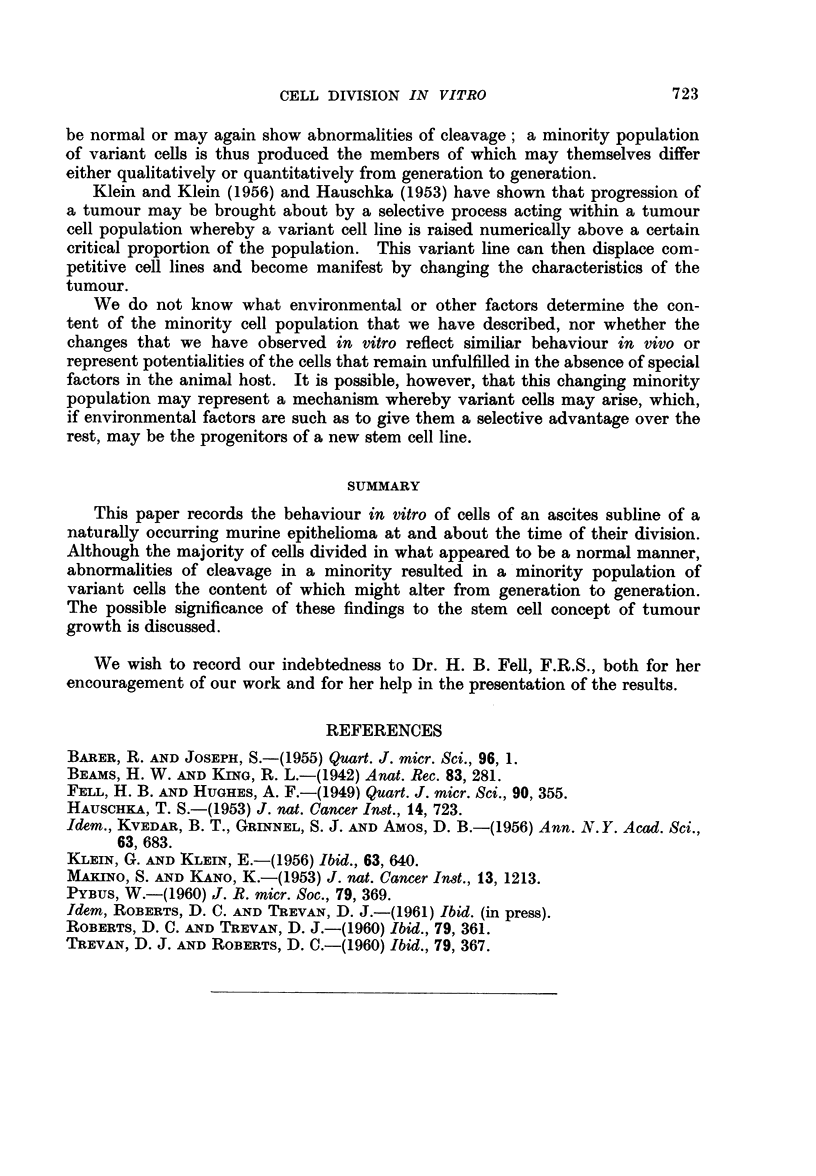# Cell Division in an Ascites Tumour in vitro

**DOI:** 10.1038/bjc.1960.81

**Published:** 1960-12

**Authors:** D. C. Roberts, D. J. Trevan

## Abstract

**Images:**


### CELL DIVISION IN AN ASCITES TUMOUR *IN VITRO*

### WITH ESPECIAL REFERENCE TO ABNORMALITIES OF CYTOPLASMIC CLEAVAGE

D. C. ROBERTS AND D. J. TREVAN

*From the Division of Experimental Biology and Virology, Imperial Cancer Research Fund, Mill Hill, London, N.W.7*

Received for publication October 1, 1960

THE concept of stem lines within populations of tumour cells is now well established, having first been put forward by Makino and Kano (1953) and by Hauschka (1953) on the basis of experiments with ascites tumours of rats and of mice respectively. According to this concept there are within certain tumour-cell populations one or more lines of stem cells which are capable of regular mitosis and which are the primary contributors to the growth of the tumour, while outside the stem lines there is a variable proportion of genetically unbalanced cells, the whole population being in a state of dynamic equilibrium which may be altered by appropriate stimuli (Hauschka *et al.*, 1956).

The purpose of this paper is to record the behaviour of cells from sublines of the derived ascites form of a naturally occurring murine epithelioma, at and about the time of their division, and to draw attention to the lability of the minority of cells in the population which divide in an abnormal manner under the conditions of our experiments.

### MATERIALS AND METHODS

The tumour cell populations used in these studies are from two derived ascites sublines of the tumour Epithelioma 255. Epithelioma 255 was found on the skin of the flank of a $2\frac{1}{4}$-year-old stock male C/57 mouse in 1955; it proved to be a well differentiated squamous cell carcinoma, and since that time has been maintained by one of us (DT) in mice of the strain of origin.

The standard culture medium consists of 16 per cent calf serum in Hanks' solution containing 0·07 per cent each of sodium bicarbonate and glucose and 0·35 per cent lactalbumin hydrolysate (" Bacto-Lactaltone ", Difco) expressed as percentages of the total volume of medium. No antibiotics are used. This medium has a pH of 7·1 when in equilibrium with 5 per cent carbon dioxide.

Before preparing a population of tumour cells for examination, all apparatus, instruments and solutions required are placed in a hot room at 36·5° C. and allowed to come to temperature. A mouse bearing an ascites subline of Epithelioma 255 is killed, pinned to a cork board and immediately transferred to the hot room in which all subsequent manipulations are done. The abdominal skin is reflected and 0·1 ml. of ascites fluid is drawn into a syringe already containing 0·9 ml. of culture medium. This is mixed and one drop is expelled into 1 ml. of culture medium. The resulting dilution (about 1 in 250) is mixed and drawn up into a second syringe.

A sample of the resulting suspension of tumour cells is introduced into a culture chamber (Roberts and Trevan, 1960) which is placed on the stage either of a cinemicroscope in the same hot room (Trevan and Roberts, 1960 ; Pybus, 1960) or of a transportable cinemicroscope (Pybus, Roberts and Trevan, 1960) also at 36·5° C. which is kept in a neighbouring laboratory. The drops in the chambers are maintained throughout the experiment in equilibrium with a water-saturated atmosphere of 5 per cent carbon dioxide in air, and the cultures are fed at appropriate intervals (usually every 1–3 days). Phase contrast optics are used on the cinemicroscopes and time-lapse cinephotomicrographic records are taken continuously throughout the course of the experiment on 16 mm. slow pan negative film at the rate of one frame every 24 or 48 seconds.

The cine records are analysed in the first instance as negatives (we develop our own film but normally have negative film printed only when we require to assemble, in one film, sequences from the records of a number of experiments), a standard 16 mm. projector with a 1 inch projector lens and a throw of 12 feet being used. We find that the angle subtended to the eye is too large for the accurate examination of the whole field of view on the screen at one time ; the screen therefore is divided into 35 squares of equal size and a film is shown either to a single observer for the requisite number of times, or once to several observers who each critically examine a part of the field.

A recent acquisition is a Muray 16 mm. film editor which, used in conjunction with a Premier footage and frame counter, is proving invaluable for the analysis of complex sequences and indispensable when events have to be timed.

### RESULTS

When first explanted and viewed on the phase microscope the cells appear as spherical bodies with no discernible internal structure surrounded by a thin rind of cytoplasm which at times shows undulating movements. Later in the experiment most of the cells flatten on the glass, and much internal structure can then be seen: nuclei, perinuclear granules and mitochondria are visible and the cytoplasm is bordered by an undulating membrane. In some circumstances the cells orientate themselves one to another and make intercellular contacts of a kind never seen between cells in the rounded form.

When a cell divides in a " normal " manner it is as follows. The cell suddenly releases all firm contacts with the glass substrate and with any surrounding cells and the cytoplasm quickly shrinks until the cell appears as a spherical body in which little or no internal structure can be seen. After a varying period of complete immobility the spherical body elongates slightly and an indentation appears on either side which deepens until two apposed daughters are formed. The cells now quite suddenly flatten out, the internal structure can again be seen, an undulating membrane reappears at the edge of the cytoplasm and once more the cells can move by their own efforts or make cohesions with their fellows according to the conditions of the experiment. In cells dividing parallel to the plane of the glass, it is common for the two daughter cells to spread out simultaneously, although sometimes one expands before the other.

An expanded cell is usually attached much more firmly to the glass substrate than a rounded cell. In the event of a cell dividing at right angles to the plane of glass, the daughter in contact with the glass completes a " normal " division ; that away from the glass, however, remains spherical with no discernible cytoplasmic movement, yet shifting as a whole in a quick and aimless manner as if wafted about by the movements of the underlying expanded cells. Immediately the spherical cell touches the glass, it flattens out.

Sometimes when a member of a sheet of cells rounds up to divide it is extruded onto the upper surface of the sheet, apparently by the pressure of the cytoplasmic borders of the surrounding, firmly attached flattened cells. Again the rounded daughter cells make no attempt to spread until they make contact with the glass substrate. Thus we can determine a period during which the cytoplasmic properties of an ascites cell differ from those of its interphase neighbours in an identical environment. This we term the functional mitotic interval : it is the period between the rounding up of a cell before division and the spreading out of a daughter cell after cleavage. The interval may conveniently be divided into two parts ; the period from the rounding up of the cell to the beginning of cytoplasmic constriction and from that constriction to the spreading of a daughter cell.

Fig. 1 shows a distribution graph of the functional mitotic intervals of the 243 cells in division that we have analysed ; we have excluded from this figure cells that lie on top of the sheet as the time at which these expand appears to be dependent on chance contact with unoccupied substrate rather than a function of the physiological state of the cells. It will be seen that the functional mitotic intervals of the cells that undergo " normal " division show a wide range of duration to produce a skew curve with a long tail in the high time range and a mode at 51–60 minutes. Although the numbers are small, it appears that the functional mitotic intervals of the cells that have been seen to divide abnormally probably follow the same pattern as those of the cells that divide " normally ".

### ABNORMALITIES OF CYTOPLASMIC CLEAVAGE

Fig. 2 indicates diagrammatically the abnormalities of division which we have seen, and roman numerals in the text refer to this figure.

The most simple abnormality of cleavage recognised was the persistence of a thread of cytoplasm joining two daughter cells when they spread out after the division of a uninucleate cell ; this thread parted to form two free daughter cells (II). In this series such cytoplasmic threads were seen on 8 occasions, and the times from the spreading out of a daughter cell to the breaking of the cytoplasmic thread varied between 18 and 43 minutes except in one cell when the thread remained for 138 minutes.

Sometimes a cytoplasmic thread failed to break ; and instead thickened until the two daughter cells came together to form a binucleate cell (III). This we have seen on 7 occasions, the time between the spreading of a daughter and the formation of a recognisable binucleate cell varying between 42 and 324 minutes. We were unable to foresee from the appearance of a thread whether it would break with the formation of two separate daughters or persist with the formation of a single binucleate cell. A similiar process was seen in 7 cells in which there was either an abortive attempt or apparently no attempt at cytoplasmic cleavage, and the cell, which was uninucleate when it rounded up, was seen to be binucleate when it re-expanded (IV).

Fig. 1.—Distribution graph of the functional mitotic intervals of ascites cells of Epithelioma 255.

Another mode of formation of a binucleate from a uninucleate cell was seen once only. A uninucleate cell rounded up to divide and cytoplasmic cleavage began 23 minutes later. Nineteen minutes after this the first daughter cell

Fig. 2.—Diagram of abnormalities of cytoplasmic cleavage seen in ascites cells of Epithelioma 255.

spread out on the glass to be followed by the second 14 minutes later. The two daughters appeared to be entirely separate from one another and despite repeated examination of the cine record no connecting thread has been seen. After a further 554 minutes however, the two cells came into close apposition and their cytoplasms fused to form a single binucleate cell (V). A binucleate cell is not necessarily committed to the binucleate state in future generations ; indeed we have not seen a binucleate cell that has produced two binucleate daughters at division. On 8 occasions we have observed binucleate cells round up to divide, undergo successful cytoplasmic cleavage and give rise to two uninucleate daughter cells (VI). On only three occasions have we seen a binucleate cell that attempted division and then again formed a single binucleate cell. In the first case cytoplasmic cleavage was nearly completed. The binucleate cell rounded up and cleavage began after 30 minutes ; the first daughter cell flattened out after a further 56 minutes, and the second 58 minutes after that. The cells were seen to be joined by a cytoplasmic thread which disappeared after 83 minutes and a single binucleate cell was again formed (VII). In the second case a binucleate cell rounded up, 62 minutes later an abortive attempt at cleavage was seen and it was not until a further 490 minutes had elapsed that the cell spread out again, still containing two nuclei (VIII). The third cell was seen to divide several times, and will be considered later.

Only once have we seen the formation of one binucleate and one uninucleate cell from the division of a single binucleate cell. This cell rounded up and after 75 minutes underwent a 3-way division. After a further 45 minutes, two of the daughter cells expanded to be followed by the third after a further 69 minutes. This third cell was seen to be joined to one of the other daughter cells by a thin cytoplasmic thread which persisted for 45 minutes ; it was then absorbed and a binucleate daughter was formed (IX).

Successive divisions each with failure of cytoplasmic cleavage give rise to very big cells with large and complex nuclear systems, although this may be followed by the death of the cell. Thus we have seen a uninucleate cell which rounded up and cytoplasmic cleavage began 14 minutes later ; cleavage was not complete and 140 minutes afterwards the cell spread out again in a binucleate state. After a further 1062 minutes the cell rounded up again and an early cytoplasmic furrow was seen after 45 minutes. Sixty-six minutes later the cell attempted to divide, failed, remained as a single cell and after another 285 minutes expanded to reveal 4 nuclei. It appeared to be quite healthy, but after a further 231 minutes the cytoplasm suddenly coagulated and it died (X).

Another cell contained two nuclei when first seen. It rounded up for the first time and a cytoplasmic furrow was visible 51 minutes later. Cytoplasmic cleavage did not take place and after a further 38 minutes it expanded again, still in a binucleate state. It remained spread for 928 minutes and then rounded up for a second time ; after 41 minutes a furrow could be seen in the cytoplasm, but there was no cytoplasmic cleavage and 60 minutes later the cell again flattened out on the glass, this time with 4 nuclei. The cell remained expanded for 1141 minutes after which it rounded up for a third time ; a cytoplasmic furrow was seen after 74 minutes followed by an attempt at a 4-way division which failed : after a further 46 minutes the cell spread out once more, still with 4 nuclei (XI).

Failure of cytoplasmic cleavage in a uninucleate cell does not preclude successfull cytoplasmic cleavage in a later generation. Twice we have seen a uninucleate cell which rounded up to divide, failed to cleave and produced a binucleate cell which subsequently divided successfully into two uninucleate daughters (XII).

### DISCUSSION

The cell preparations used in this study were prepared in such a way as to give the best possible visualisation of the cytoplasm. The refractive index of the culture medium was too low to permit analysis of the behaviour of the chromosomes during cell division, and we do not propose to discuss here the effects on ploidy that may result from these aberrant cell divisions. The examination of cells in a high protein medium as described by Barer and Joseph (1955) might well allow the nuclear behaviour of similar cells to be studied and correlations determined. Comparisons could then be made with the modes of formation of binucleate and polyploid cells in non-neoplastic tissues as described in the regenerating liver of the rat by Beams and King (1942) and in cultures of mouse fibroblasts by Fell and Hughes (1949).

The picture that emerges from this *in vitro* study is that of a population of cells in which the great majority of cells that divide do so in an apparently normal manner while a minority show a wide range of abnormalities of cytoplasmic cleavage. The cells resulting from these abnormal divisions are usually viable and have frequently been seen to undergo further division which may appear to

---

### EXPLANATION OF PLATES

FIG. 3–5.—Formation of a binucleate cell from a uninucleate cell.
- FIG. 3.—A uninucleate cell (A) before division. × 280.
- FIG. 4.—Daughter cells joined by a cytoplasmic thread. × 280.
- FIG. 5.—The thread has been absorbed with the formation of a single binucleate cell. × 280.

FIG. 6–7.—Formation of a binucleate cell from a uninucleate cell.
- FIG. 6.—A uninucleate cell (B) before division. × 280.
- FIG. 7.—Resulting binucleate cell from (B). × 280.

FIG. 8–11.—Formation of a binucleate cell from a uninucleate cell.
- FIG. 8.—Uninucleate cell (C) before division. × 330.
- FIG. 9.—Two apparently separate daughters (D) and (E) from the division of (C). × 330.
- FIG. 10.—Fusion taking place between (D) and (E). × 330.
- FIG. 11.—A binucleate cell (G) resulting from the fusion of (D) and (E). × 330.

FIG. 12–15.—A uninucleate cell which divides to form a single binucleate cell. This cell then divides to form two uninucleate daughters.
- FIG. 12.—Uninucleate cell (H) before division. × 330.
- FIG. 13.—Binucleate cell (I) resulting from division of (H). × 330.
- FIG. 14.—Binucleate cell (I) just before its division. × 330.
- FIG. 15.—Two uninucleate cells (J) and (K) resulting from the division of (I). × 330.

FIG. 16–19.—Formation of one uninucleate and one binucleate daughter from the division of a single binucleate cell.
- FIG. 16.—Binucleate cell (L) before division. × 280.
- FIG. 17.—Three-way division of cell (L). × 280.
- FIG. 18.—Daughters from division of cell (L). Two daughters are expanded while one is still rounded up. × 280.
- FIG. 19.—Binucleate cell (M) and uninucleate cell (N) formed from the division of cell (L). × 280.

FIG. 20–23.—Formation of cell with complex nuclear structure from a binucleate cell.
- FIG. 20.—Binucleate cell before division. × 330.
- FIG. 21.—The cell has attempted division, there has been failure of cytoplasmic cleavage, and a binucleate cell is again formed. × 330.
- FIG. 22.—Attempted division has again taken place; cleavage has again failed and a single 4-nucleate cell has been formed. Only three of the nuclei are visible in this figure. × 330.
- FIG. 23.—The 4-nucleate cell has attempted division, cleavage has again failed, with the formation again of a cell with 4 nuclei. × 330.

3

4

5

6

7

Roberts and Trevan.

8

9

10

11

12

13

14

15

Roberts and Trevan.

16

17

18

19

20

21

22

23

be normal or may again show abnormalities of cleavage ; a minority population of variant cells is thus produced the members of which may themselves differ either qualitatively or quantitatively from generation to generation.

Klein and Klein (1956) and Hauschka (1953) have shown that progression of a tumour may be brought about by a selective process acting within a tumour cell population whereby a variant cell line is raised numerically above a certain critical proportion of the population. This variant line can then displace competitive cell lines and become manifest by changing the characteristics of the tumour.

We do not know what environmental or other factors determine the content of the minority cell population that we have described, nor whether the changes that we have observed *in vitro* reflect similiar behaviour *in vivo* or represent potentialities of the cells that remain unfulfilled in the absence of special factors in the animal host. It is possible, however, that this changing minority population may represent a mechanism whereby variant cells may arise, which, if environmental factors are such as to give them a selective advantage over the rest, may be the progenitors of a new stem cell line.

### SUMMARY

This paper records the behaviour *in vitro* of cells of an ascites subline of a naturally occurring murine epithelioma at and about the time of their division. Although the majority of cells divided in what appeared to be a normal manner, abnormalities of cleavage in a minority resulted in a minority population of variant cells the content of which might alter from generation to generation. The possible significance of these findings to the stem cell concept of tumour growth is discussed.

We wish to record our indebtedness to Dr. H. B. Fell, F.R.S., both for her encouragement of our work and for her help in the presentation of the results.

### REFERENCES

Barer, R. and Joseph, S.—(1955) *Quart. J. micr. Sci.*, **96**, 1.
Beams, H. W. and King, R. L.—(1942) *Anat. Rec.* **83**, 281.
Fell, H. B. and Hughes, A. F.—(1949) *Quart. J. micr. Sci.*, **90**, 355.
Hauschka, T. S.—(1953) *J. nat. Cancer Inst.*, **14**, 723.
*Idem.*, Kvedar, B. T., Grinnel, S. J. and Amos, D. B.—(1956) *Ann. N.Y. Acad. Sci.*, **63**, 683.
Klein, G. and Klein, E.—(1956) *Ibid.*, **63**, 640.
Makino, S. and Kano, K.—(1953) *J. nat. Cancer Inst.*, **13**, 1213.
Pybus, W.—(1960) *J. R. micr. Soc.*, **79**, 369.
*Idem*, Roberts, D. C. and Trevan, D. J.—(1961) *Ibid.* (in press).
Roberts, D. C. and Trevan, D. J.—(1960) *Ibid.*, **79**, 361.
Trevan, D. J. and Roberts, D. C.—(1960) *Ibid.*, **79**, 367.